# Comparison of clinical outcomes between mobile- and fixed-bearing unicompartmental knee arthroplasty for medial compartment osteoarthritis: a retrospective study

**DOI:** 10.3389/fsurg.2026.1746302

**Published:** 2026-09-28

**Authors:** Hongze Chen, Xiaodong Zheng, Shaobin Zhu, Longyu Shan, Jixin Chen, Lei Wan, Feng Ye

**Affiliations:** Department of Orthopaedic Surgery, Shaoxing Shangyu District Hospital of Traditional Chinese Medicine, Shaoxing, China

**Keywords:** fixed-bearing, medial osteoarthritis, mobile-bearing, range of motion, unicompartmental knee arthroplasty

## Abstract

**Background:**

Unicompartmental knee arthroplasty (UKA) is an established treatment for isolated medial compartment osteoarthritis (MCOA). The optimal choice between mobile-bearing (MB) and fixed-bearing (FB) designs remains debated, particularly in Asian populations where treatment costs and insurance policies influence surgical decisions.

**Objective:**

To compare short-term clinical and radiographic outcomes between MB and FB UKA and to evaluate potential implications for healthcare resource allocation.

**Methods:**

Sixty-four patients who underwent medial UKA between January 2022 and October 2024 were retrospectively analyzed (MB: *n* = 32; FB: *n* = 32). All surgeries were performed by a single senior surgeon using standardized protocols. Pain (Visual Analogue Scale, VAS), function (Hospital for Special Surgery [HSS] and American Knee Society Score [KSS]), radiographic alignment (femorotibial angle, FTA; tibial slope angle, STA), and range of motion (ROM) were assessed preoperatively and at 3 months postoperatively. Statistical significance was set at *P* < 0.05.

**Results:**

Both groups achieved significant improvements in VAS, HSS, and KSS scores (all *P* < 0.001), with no intergroup differences. MB showed greater correction in FTA (−3.27° vs. −2.70°, *P* < 0.05) and larger ROM gains in flexion and extension compared with FB (*P* < 0.05). No infections, loosening, or bearing dislocations occurred.

**Conclusions:**

MB and FB UKA provide comparable short-term pain relief and functional improvement. MB may offer greater alignment correction and range of motion, whereas FB was associated with lower hospitalization cost in this cohort and may therefore have a potential economic advantage. Prosthesis selection should consider both clinical performance and economic factors to optimize patient outcomes and resource utilization.

## Introduction

Knee osteoarthritis (KOA) is a common chronic degenerative disease whose prevalence increases with age and is one of the leading causes of disability worldwide. With the aging population, the prevalence of KOA is steadily rising, resulting in significant public health and socioeconomic burdens (1, 2). The medial compartment of the knee is the most commonly affected, and patients with isolated medial compartment osteoarthritis (MCOA) often suffer from chronic pain, joint deformity, and limited mobility, severely impairing their quality of life (3).

Unicompartmental knee arthroplasty (UKA) has emerged as an effective surgical alternative to total knee arthroplasty (TKA) for patients with isolated medial compartment osteoarthritis. Compared to TKA, UKA preserves more native bone and soft tissue, retains the cruciate ligaments, and allows for faster rehabilitation and more physiological knee kinematics. These advantages contribute to quicker functional recovery, reduced perioperative morbidity, and satisfactory long-term implant survival rates (4). Thus, UKA plays an important role in improving patients’ postoperative quality of life (5). UKA prostheses are generally classified into two types: fixed-bearing (FB) and mobile-bearing (MB). The MB design allows the polyethylene insert to move relative to the tibial baseplate, reducing contact stress and polyethylene wear while closely mimicking natural knee motion. In contrast, the FB design features a fixed polyethylene insert that is locked to the tibial tray, offering improved implant stability and minimizing the risk of bearing dislocation (6, 7). National joint registries provide valuable real-world evidence on the long-term survivorship of UKA prostheses, yet they have notable limitations: different implant designs and surgical techniques are often pooled together in analysis, which may mask the performance characteristics of specific prosthesis models. The Orthopaedic Data Evaluation Panel (ODEP) provides standardized rating of implant survival data based on published clinical evidence. The ZUK fixed-bearing prosthesis (Enovis) has been awarded the highest 15A* ODEP rating, confirming its excellent long-term clinical performance. The Zimmer fixed-bearing prosthesis used in our study also has favorable long-term survival data reported in multiple international registries and holds a high ODEP rating, supporting its reliable clinical application. Although both designs have been successfully implemented in clinical practice, the optimal choice remains debated due to differences in kinematics, technical complexity, and long-term survivorship. Existing clinical studies and meta-analyses have reported inconsistent findings, with some suggesting similar outcomes in pain relief, knee function, and revision rates, while others highlight differences in alignment correction or polyethylene wear (6, 8). Compared with fixed-bearing designs, mobile-bearing UKA carries a slightly higher risk of polyethylene bearing dislocation. According to recent meta-analyses and large-scale registry studies, the overall incidence of bearing dislocation in modern mobile-bearing UKA ranges from 0.5% to 2.0%, and this risk can be effectively minimized through accurate intraoperative component alignment, proper soft tissue balancing, and standardized patient selection. However, most of these studies have focused on Western populations and involved heterogeneous surgical techniques and prosthesis types, limiting their generalizability. Furthermore, there is limited evidence regarding the early postoperative outcomes of MB and FB UKA in Chinese patients undergoing standardized surgical techniques.

Therefore, this study aims to compare the short-term clinical efficacy of MB and FB UKA for MCOA, with a focus on pain relief, functional recovery, lower limb alignment, and range of motion. We hypothesize that both designs will provide comparable pain relief and functional recovery, with the MB design potentially offering an advantage in restoring physiological lower limb alignment.

## Methods

### Study design and participants

This retrospective study was conducted at Shangyu Traditional Chinese Medicine Hospital from January 2022 to October 2024 to compare the short-term clinical outcomes of MB and FB UKA for MCOA. A total of 64 patients diagnosed with isolated medial compartment osteoarthritis of the knee were included and divided into two groups: the MB group (*n* = 32) and the FB group (*n* = 32). All surgeries were performed by the same senior orthopedic surgeon to minimize variability in surgical technique. Baseline characteristics, including age, sex, body mass index (BMI), preoperative knee flexion angle, and the affected side, were comparable between the two groups (*P* > 0.05), ensuring a balanced comparison. This study was approved by the institutional ethics committee, and written informed consent was obtained from all participants.

### Inclusion and exclusion criteria

Patients were included if they had isolated medial compartment osteoarthritis confirmed by clinical and radiographic evaluation, experienced medial knee pain as the primary symptom, and met the established indications for unicompartmental knee replacement. No specific chronological age cutoff was applied; age alone was not used as an inclusion or exclusion criterion. Exclusion criteria included severe systemic disease or contraindications to surgery, severe osteoporosis (T-score < −2.5), infection at the knee or other sites, and evidence of multi-compartmental knee arthritis or cruciate ligament insufficiency on radiographic or intraoperative assessment.

### Surgical procedures

All patients underwent standardized preoperative preparation, including routine blood tests, radiographic evaluation, and fasting prior to surgery. Under general anesthesia and tourniquet control, a medial parapatellar incision approximately 8–10 cm in length was made to expose the medial compartment. For the FB group, a cemented FB prosthesis was implanted. Tibial resection was performed using an extramedullary guide, with a resection thickness of approximately 4 mm, followed by femoral condyle resection and balancing of flexion–extension gaps. After trial reduction confirmed satisfactory stability and range of motion, the tibial and femoral components were cemented sequentially, and excess cement was removed before polymerization (Figure 1). For the MB group, the Oxford MB prosthesis was used. After removing osteophytes and performing notch preparation, vertical and horizontal tibial cuts were made using extramedullary alignment. Femoral condyle shaping was conducted according to the prosthesis size. Trial components were inserted to confirm proper ligament tension and gap balance, after which final cementation was performed. The polyethylene bearing was inserted with the knee flexed at 45°, and the limb was maintained in this position until cement fixation was complete (Figure 2). In both groups, 1 g of tranexamic acid was administered intravenously 30 min before incision. A periarticular cocktail injection (100 mg ropivacaine + 40 mg methylprednisolone + 40 mL saline) was used for local infiltration anesthesia. No drainage tube was placed postoperatively. Antibiotic prophylaxis with ceftriaxone and anticoagulation with rivaroxaban were provided. Rehabilitation training began on postoperative day 2 under physiotherapist supervision, and partial weight-bearing ambulation was initiated on postoperative day 3.

**Figure 1:**
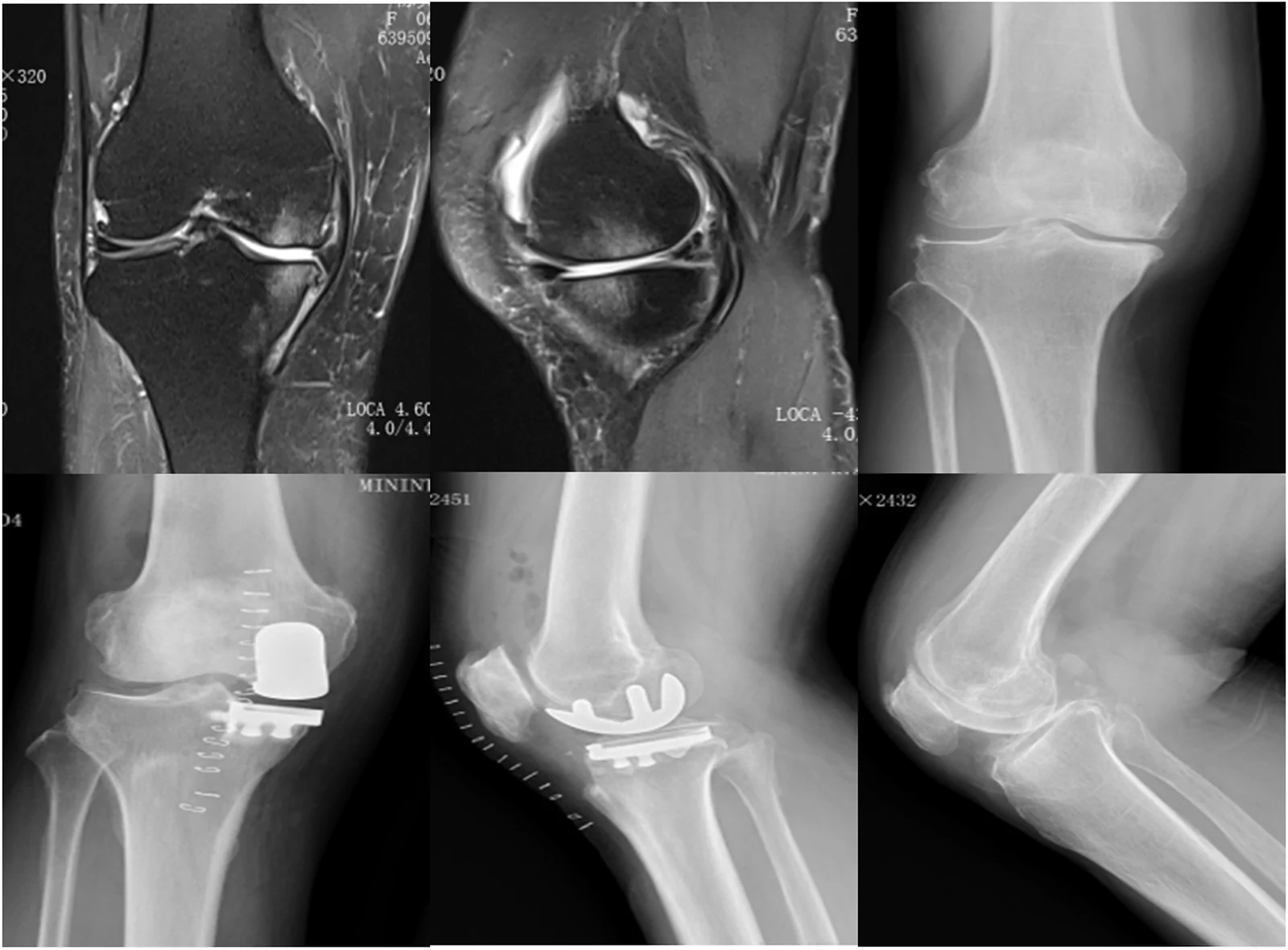
Representative case of fixed-bearing UKA. A 70-year-old female patient underwent medial unicompartmental knee arthroplasty with a fixed-bearing prosthesis for medial compartment osteoarthritis of the right knee. At the 24-month routine clinical follow-up, the patient reported satisfaction with the treatment outcome and no significant discomfort. This later observation is presented for illustration only and was not included in the comparative statistical analysis, which was limited to the 3-month endpoint.

**Figure 2:**
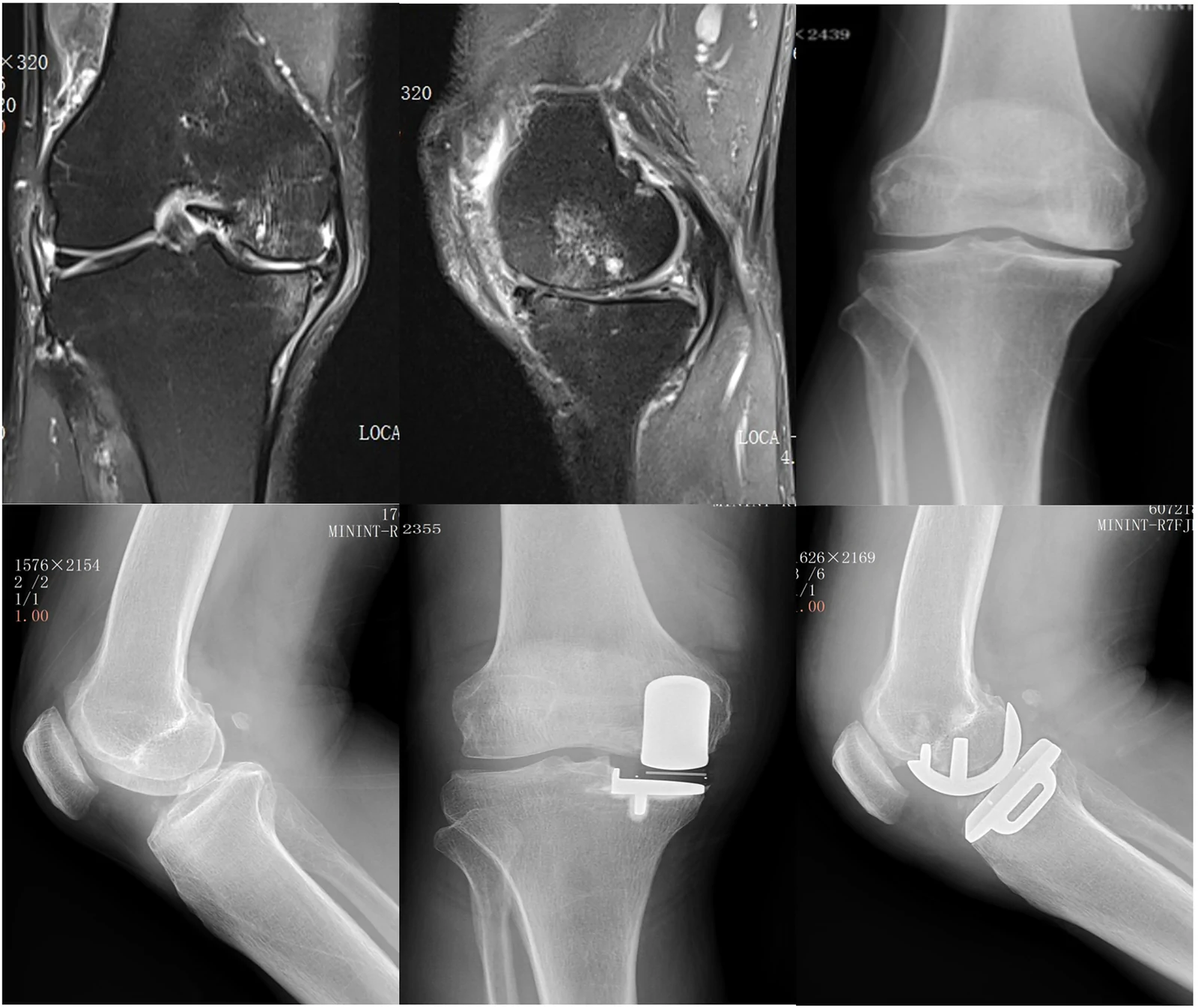
Representative case of mobile-bearing UKA. A 69-year-old male patient underwent medial unicompartmental knee arthroplasty with a mobile-bearing prosthesis for medial compartment osteoarthritis of the right knee. At the 31-month routine clinical follow-up, the patient demonstrated satisfactory knee function. This later observation is presented for illustration only and was not included in the comparative statistical analysis, which was limited to the 3-month endpoint.

### Outcome measures

Clinical and radiological outcomes used for the comparative analysis were assessed preoperatively and at 3 months postoperatively. The later routine follow-up observations shown for the two representative cases in Figures 1,2 (24 and 31 months, respectively) are illustrative only and were not included in the comparative statistical analysis. Pain intensity was evaluated using the Visual Analogue Scale (VAS). Knee function was assessed with the Hospital for Special Surgery (HSS) score and the American Knee Society Score (KSS). Radiographic measurements included the femorotibial angle (FTA) and the tibial slope angle (STA), recorded from standardized anteroposterior and lateral radiographs. The maximum flexion and extension angles of the knee were also measured. Intraoperative parameters, including surgery time and intraoperative blood loss, as well as length of hospital stay and hospitalization cost, were recorded. Postoperative complications, including infection, prosthesis loosening, and bearing dislocation, were documented.

### Statistical analysis

Statistical analysis was performed using SPSS version 26.0 (IBM Corp., Armonk, NY, USA). Continuous data were expressed as mean ± standard deviation (SD). Between-group comparisons were conducted using independent-sample t-tests, and within-group comparisons (preoperative vs. postoperative) were conducted using paired-sample t-tests. Categorical variables were compared using the chi-square test. A *P* value of < 0.05 was considered statistically significant.

## Results

A total of 64 patients (32 in the MB group and 32 in the FB group) underwent surgery without intraoperative complications; no infections, implant loosening, or bearing dislocations were observed during the 3-month comparative follow-up. Baseline variables—age, sex, body mass index (BMI), preoperative knee flexion, and affected side—were comparable between groups (*P* > 0.05), confirming baseline homogeneity. In the MB group, there were 19 men and 13 women; the mean age was 70.06 ± 5.58 years (range 54–80 years), mean BMI was 22.78 ± 4.05 kg/m^2^, mean preoperative flexion was 116.43 ± 5.56°, and 20 right and 12 left knees were treated. The FB group comprised 20 men and 12 women; the mean age was 70.17 ± 5.42 years (range 53–86 years), mean BMI was 23.02 ± 3.68 kg/m^2^, mean preoperative flexion was 116.79 ± 5.78°, and 20 right and 12 left knees were treated.

### Pain assessment

Both groups showed significant reductions in pain following surgery. In the MB group, the mean VAS score decreased from 5.98 ± 0.52 preoperatively to 1.20 ± 0.20 at three months postoperatively (*P* < 0.001). Similarly, the FB group exhibited a decrease in VAS score from 5.95 ± 0.56 preoperatively to 1.23 ± 0.25 postoperatively (*P* < 0.001). No significant difference in postoperative VAS scores was found between the two groups (*P* > 0.05) (Table 1).

**Table 1:** Comparison of pain scores between the two groups before and after surgery (X̄ ± S, points).

Group	*n*	Preoperative	3 months postoperative	t	*P*
Activity group	32	5.98 ± 0.52	1.20 ± 0.20	48.235	<0.001
Fixation group	32	5.95 ± 0.56	1.23 ± 0.25	48.327	<0.001
t		0.412	0.127		
*P*		0.676	0.856		

### Functional outcomes

Both groups demonstrated substantial improvements in knee function. In the MB group, the mean KSS increased from 48.03 ± 5.20 preoperatively to 87.30 ± 8.30 postoperatively (*P* < 0.001), and the HSS score improved from 64.03 ± 4.20 to 91.30 ± 2.30 (*P* < 0.001). The FB group also showed marked improvements, with the KSS rising from 49.01 ± 5.10 to 85.04 ± 8.10 (*P* < 0.001), and the HSS score increasing from 62.01 ± 5.50 to 90.04 ± 2.12 (*P* < 0.001). However, no significant differences were observed between the groups in both the KSS and HSS scores postoperatively (*P* > 0.05) (Tables 2, 3).

**Table 2:** Comparison of KSS scores between the two groups before and after surgery (X̄ ± S, points).

Group	*n*	Preoperative	3 months postoperative	t	*P*
Activity group	32	48.03 ± 5.20	87.30 ± 8.30	22.022	<0.001
Fixation group	32	49.01 ± 5.10	85.04 ± 8.10	22.301	<0.001
t		0.086	0.039		
*P*		0.921	0.946		

**Table 3:** Comparison of HSS scores between the two groups before and after surgery (X̄ ± S, points).

Group	*n*	Preoperative	3 months postoperative	t	*P*
Activity group	32	64.03 ± 4.20	91.30 ± 2.30	27.022	<0.001
Fixation group	32	62.01 ± 5.50	90.04 ± 2.12	26.508	<0.001
t		0.056	0.170		
*P*		0.976	0.846		

### Radiographic parameters and alignment correction

Both the FTA and STA showed significant reductions after surgery, indicating effective correction of lower limb alignment. In the MB group, the FTA decreased from 179.15 ± 3.16° preoperatively to 175.88 ± 3.22° postoperatively (*P* = 0.002), and the STA decreased from 4.05 ± 0.36° to 1.84 ± 0.32° (*P* = 0.011). In the FB group, the FTA decreased from 178.88 ± 3.24° to 176.18 ± 3.25° (*P* = 0.001), and the STA decreased from 4.06 ± 0.38° to 1.83 ± 0.41° (*P* < 0.001).

Furthermore, the MB group exhibited a significantly greater improvement in alignment correction, with a greater reduction in both FTA and STA compared to the FB group (*P* < 0.05). These results suggest that the MB design may be more effective in restoring lower limb alignment, as indicated by the more substantial reduction in the femorotibial and tibial slope angles (Table 4).

**Table 4:** Comparison of FTA, STA, maximum flexion, and maximum extension angles before and after surgery between the two groups.

Group	FTA (°)				STA (°)				Maximum flexion angle (°)				Maximum extension angle (°)			
	Pre-op	3 mo Post-op	t	*P*	Pre-op	3 mo Post-op	t	*P*	Pre-op	3 mo Post-op	t	*P*	Pre-op	3 mo Post-op	t	*P*
Activity group	179.15 ± 3.16	175.88 ± 3.22	5.901	0.002	4.05 ± 0.36	1.84 ± 0.32	48.041	0.011	124.4 ± 9.21	130.3 ± 9.99	2.737	0.011	3.46 ± 0.87	1.54 ± 0.62	17.999	0.014
Fixation group	178.88 ± 3.24	176.18 ± 3.25	4.620	0.001	4.06 ± 0.38	1.83 ± 0.41	44.500	0.000	124.9 ± 9.86	128.1 ± 10.13	1.514	0.017	3.56 ± 0.77	1.63 ± 0.73	16.036	0.001

### Range of motion

Both groups demonstrated significant improvements in knee range of motion postoperatively. In the MB group, the maximum knee flexion angle increased from 124.4 ± 9.21° preoperatively to 130.3 ± 9.99° at three months postoperatively (*P* = 0.011), while the maximum knee extension angle improved from 3.46 ± 0.87° to 1.54 ± 0.62° (*P* = 0.014). Similarly, in the FB group, the maximum flexion angle increased from 124.9 ± 9.86° to 128.1 ± 10.13° (*P* = 0.017), and the maximum extension angle improved from 3.56 ± 0.77° to 1.63 ± 0.73° (*P* = 0.001). Intergroup comparison revealed a statistically significant advantage in range of motion for the MB group, with a greater improvement in both flexion and extension angles (*P* < 0.05) (Table 4).

### Intraoperative parameters and postoperative outcomes

The comparison of intraoperative parameters and postoperative outcomes between the two groups is presented in Table 5. Surgery time in the MB group was significantly longer than in the FB group, with mean values of 79.23 ± 8.45 min and 68.94 ± 7.62 min, respectively. The difference between the groups was statistically significant (t = 5.324, *P* < 0.001). There was no significant difference in intraoperative blood loss between the two groups. The MB group had a mean blood loss of 51.34 ± 7.82 mL, while the FB group had 48.72 ± 6.95 mL. The difference was not statistically significant (t = 1.628, *P* = 0.108). Hospitalization costs differed significantly between the two groups. The MB group incurred higher costs of 34,127 ± 3,258 RMB compared with 32,164 ± 2,891 RMB in the FB group (t = 2.785, *P* = 0.007), corresponding to an average difference of approximately 1,963 RMB. Length of hospital stay did not significantly differ between the two groups. The MB group had a mean stay of 12.03 ± 1.85 days, while the FB group had 11.88 ± 1.71 days (t = 0.372, *P* = 0.711).

**Table 5:** Comparison of intraoperative parameters and postoperative outcomes between the active platform group and the fixed platform group.

Group	Surgery time (min)	Intraoperative blood loss (mL)	Length of hospital stay (days)	Hospital cost (RMB)
Mobile-bearing Group	79.23 ± 8.45	51.34 ± 7.82	12.03 ± 1.85	34,127 ± 3,258
Fixed-bearing Group	68.94 ± 7.62	48.72 ± 6.95	11.88 ± 1.71	32,164 ± 2,891
t Value	5.324	1.628	0.372	2.785
*P* Value	< 0.001	0.108	0.711	0.007

### Postoperative complications

No cases of postoperative complications, including infection, prosthesis loosening, or bearing dislocation, were observed in either group throughout the follow-up period. The absence of such complications underscores the safety and reliability of both prosthesis types when performed by experienced surgeons using standardized surgical techniques.

## Discussion

This study compared the short-term clinical outcomes of MB and FB UKA in patients with medial compartment osteoarthritis. Both prosthesis designs resulted in significant improvements in pain relief, knee function, and lower limb alignment. However, the MB design demonstrated a greater improvement in alignment correction and range of motion compared to the FB design. Despite these differences, both groups showed comparable postoperative pain scores and functional outcomes, with no significant differences in the HSS and KSS.

### Pain and functional outcomes

The results of this study are consistent with previous research, which has shown that both MB and FB UKA provide excellent short-term pain relief and functional recovery. The significant reduction in pain (measured by VAS) and substantial improvements in knee function (measured by KSS and HSS) in both groups highlight the effectiveness of UKA in managing medial compartment osteoarthritis (7 9, 10). Our findings suggest that both prosthesis designs are equally capable of alleviating pain and improving knee function in the early postoperative period, which is consistent with other studies in Western populations (11).

### Radiographic alignment and range of motion

Although both prosthesis designs resulted in significant improvements in lower limb alignment, the MB group demonstrated a more substantial correction in both the FTA and STA. This suggests that the MB design may have an advantage in restoring the physiological alignment of the knee joint, likely due to its ability to adapt to soft tissue forces and reduce stresses at the bone-implant interface (11, 12). The rotational mobility of the MB insert may allow for better accommodation of soft tissue tension, which plays a critical role in maintaining proper alignment and stability. In contrast, while the FB design provides excellent implant stability, it limits the rotational movement of the tibial and femoral components, potentially restricting the correction of varus deformities. Furthermore, the MB group exhibited superior improvements in both knee flexion and extension compared to the FB group, a finding that may be attributed to the more natural kinematic motion provided by the MB design (8). These results align with previous studies suggesting that MB UKA may offer advantages in range of motion. The FB group demonstrated a significantly shorter surgery time than the MB group; however, intraoperative blood loss did not differ significantly between groups (*P* = 0.108), as shown in Table 5. Therefore, any procedural efficiency suggested by our data relates to operative time rather than blood loss. Moreover, no significant differences in postoperative complications were observed, while the MB group showed a greater improvement in knee range of motion (13).

### Hospitalization cost and health insurance considerations

While the MB design showed greater improvements in alignment and range of motion, the FB group had a significantly lower hospitalization cost in this cohort (32,164 ± 2,891 RMB vs. 34,127 ± 3,258 RMB; *P* = 0.007). This observed difference may represent a potential economic advantage of FB UKA in settings where treatment cost influences prosthesis selection. However, because our analysis was limited to hospitalization cost, these findings should not be interpreted as evidence of formal cost-effectiveness (14).

In healthcare systems with public insurance or constrained resources, hospitalization cost can be an important consideration in treatment selection. The lower hospitalization cost observed in the FB group may improve economic feasibility for some patients and institutions. From a policy perspective, the potential clinical advantages of MB in alignment correction and range of motion should be weighed against the observed difference in hospitalization cost. In this context, FB may offer a reasonable alternative with comparable short-term pain relief and functional improvement, while formal economic evaluations are still needed to determine cost-effectiveness (15).

### Postoperative complications and safety

Both groups in this study experienced no major postoperative complications, such as infection, prosthesis loosening, or bearing dislocation, throughout the 3-month follow-up period. This highlights the safety of both prosthesis types when used with standardized surgical techniques and appropriate patient selection (16, 17). While MB UKA carries a slightly higher risk of bearing dislocation, as has been noted in some studies, careful intraoperative technique and proper alignment can mitigate this risk.

## Limitations and future directions

This study has several limitations. First, it is a single-center retrospective study with a relatively small sample size, which may limit the generalizability of the findings. Second, the comparative follow-up period was only 3 months; the later observations shown in Figures 1,2 are illustrative case follow-ups and were not included in the comparative analysis. Longer-term comparative data are needed to assess prosthesis survival, wear, and late complications. Third, the study did not assess patient satisfaction or quality of life, which are important patient-reported outcomes after knee arthroplasty. Fourth, only hospitalization cost was compared; a formal cost-effectiveness analysis incorporating longer-term costs, quality-adjusted life-years, or incremental cost-effectiveness ratios was not performed. Therefore, the economic findings should be interpreted as a difference in hospitalization cost rather than definitive evidence of cost-effectiveness. Future studies with larger sample sizes, longer follow-up, patient-reported outcomes, and formal health-economic evaluation are needed to provide more comprehensive evidence regarding MB and FB UKA.

## Conclusion

Both MB and FB UKA designs are effective in relieving pain, improving function, and restoring alignment in patients with medial compartment osteoarthritis. In this cohort, MB showed greater improvements in alignment correction and range of motion, whereas FB was associated with a lower hospitalization cost and may therefore offer a potential economic advantage in resource-constrained settings. Because no formal cost-effectiveness analysis was performed, prosthesis selection should be guided by clinical characteristics, surgeon expertise, patient needs, and healthcare resource considerations rather than by definitive cost-effectiveness claims.

## Data Availability

The raw data supporting the conclusions of this article will be made available by the authors, without undue reservation.
